# Detecting Polarized Side-Scattering Signals in Media with Ultra-Low-Scattering Coefficients: An Improved Monte Carlo Simulation Approach

**DOI:** 10.3390/s26072105

**Published:** 2026-03-28

**Authors:** Chenyu Shan, Lin He, Bingjie Jin, Zhengbang Wu, Shihe Yi

**Affiliations:** College of Aerospace Science and Technology, National University of Defense Technology, Changsha 410073, China; shanchenyu@nudt.edu.cn (C.S.); jinbingjie24@nudt.edu.cn (B.J.); wuzhengbang20@nudt.edu.cn (Z.W.); yishihe@nudt.edu.cn (S.Y.)

**Keywords:** low-scattering media, side-scattering, degree of linear polarization, polarized Monte Carlo simulation, backward ray tracing

## Abstract

**Highlights:**

**What are the main findings?**

- A novel polarized Monte Carlo algorithm integrating backward ray tracing with geometric projection correction is proposed to address the geometric mismatch in side-scattering simulations for low-scattering coefficient media ($\mu_{s}$ ≤ 1 cm^−1^).
- Experimental validation using 500 nm polystyrene microspheres and 5 nm TiO_2_ nanoparticles under 532 nm laser illumination shows that the proposed method accurately reproduces the spatial distribution of scattered light and the monotonic decrease in the degree of linear polarization with increasing concentration in ultra-low-scattering regimes ($\mu_{s}$ ~ 10^−5^ cm^−1^).

**What are the implications of the main findings?**

- This work extends the applicability of polarized Monte Carlo simulations to media with scattering coefficients as low as 10^−5^ cm^−1^, providing a reliable tool for studying light scattering in dilute particulate systems.
- The proposed algorithm enhances simulation fidelity in scenarios relevant to nanoparticle characterization, biomedical imaging, and flow diagnostics, where low-scattering conditions are prevalent.

**Abstract:**

Polarized side-scattering techniques are widely used in aerosol detection, oceanographic optics, and biomedical sensing due to their high sensitivity to weak optical signals in low-scattering coefficient media. Conventional polarized Monte Carlo methods face significant challenges in such regimes due to geometric mismatch, where photon exit positions deviate substantially from the detector plane. This study addresses the geometric mismatch issue in polarized Monte Carlo simulations for side scattering in low-scattering media (scattering coefficient $\mu_{s}=$ 1 cm^−1^), where photon exit positions often deviate from the detector plane. We propose a novel algorithm incorporating backward ray tracing with geometric projection correction to enhance simulation accuracy. Experimental validation was conducted using 532 nm laser illumination on both 500 nm polystyrene microspheres ($\mu_{s}=$ 0.21 cm^−1^) and 5 nm TiO_2_ nanoparticles ($\mu_{s}=$ 1.06 × 10^−6^–1.06 × 10^−5^ cm^−1^). The results demonstrate excellent agreement between simulations and experiments, confirming the algorithm’s capability to accurately capture the polarization characteristics of side-scattered light. This work provides a high-fidelity simulation tool for designing optical sensors in low-scattering media and holds direct applicability in nanoparticle concentration sensing and aerosol monitoring.

## 1. Introduction

The non-invasive and high-resolution nature of polarized laser side scattering makes it indispensable for medical diagnosis [1,2], atmospheric measurement [3,4], particle concentration measurement [5,6], flow field imaging [7,8,9] and aerospace propulsion plume diagnostics [10,11]. For instance, Phan and Lo [12] utilized polarized laser side scattering to determine glucose concentration in solution. Porcar et al. [13] used polarization-resolved side scattering to show how wall injection alters the internal flow of a free jet under non-induced conditions. He et al. [14] employed polarization imaging to visualize shock-wave/boundary-layer interactions (SWBLI) in supersonic flows, demonstrating that the degree of linear polarization directly maps the concentration and orientation of tracer particles in the measurement volume. Across all these scenarios, the unifying objective is to quantify the polarized side-scattered light from particles in the medium. This observable enables reliable extraction of particle concentration, media composition, and flow-field dynamics.

Many of these applications, particularly in the detection of atmospheric aerosols and oceanic particulates, involve media with very low particle concentrations, resulting in low-scattering coefficients. In atmospheric measurement, particle concentration measurement, and flow field imaging, low-scattering coefficient (scattering coefficient $\mu_{s}<$ 1 cm^−1^) polarized side-scattered light is crucial. These scenarios typically involve low particle concentrations, which result in a low-scattering coefficient. This makes studying low-scattering coefficient polarized side-scattered light highly significant. However, current research predominantly focuses on high-scattering coefficient (scattering coefficient $\mu_{s}\geq$ 1 cm^−1^) scenarios, leaving low-scattering coefficient research relatively underexplored.

For high-scattering coefficients, researchers have extensively investigated polarized scattered light through simulations and experiments. On the simulation side, Monte Carlo methods, renowned for their effectiveness in handling complex and multiple scattering events, have become the general algorithm for simulating polarized light propagation. The three polarized Monte Carlo frameworks proposed by Ramella-Roman et al. successfully model high-scattering coefficient media by tracking photon paths and Stokes vector evolution [15,16]. Bartel et al. [17] developed a light scattering polarization program, presenting simulation and experimental diagrams of back-scattering Mueller matrices for 204 nm diameter polystyrene suspended particles. Tian et al. [18] showed via Monte Carlo simulations that linear and circular polarizations give equal image quality for small scatterers, whereas circular polarization outperforms for larger ones in isotropic media. On the experimental side, Yuan et al. [19] measured side-scattered polarization from microparticle solutions at varied angles and incident states, quantifying intensity and degree of linear polarization (DoLP) trends. Wu et al. [20] found that nanoparticles and microparticles exhibit distinct side-scattering polarization, almost independent of incident intensity.

The above investigations have exclusively focused on media characterized by high-scattering coefficients. In low (scattering coefficient $\mu_{s}<$ 1 cm^−1^) and ultra-low (scattering coefficient $\mu_{s}<$ 10^−4^ cm^−1^) scattering coefficient regimes, two critical problems for conventional Monte Carlo methods emerge: (1) geometric mismatch: fixed-boundary assumptions and significant increase in photon mean free path (>10 cm) cause photon exit positions to deviate from the detector plane, inducing lateral positional errors with magnitudes reaching centimeters, severely degrading simulation accuracy; (2) failure in low $\mu_{s}$ regimes: For $\mu_{s}<$ 1 cm^−1^, the probability of photon escape from the detection zone increases significantly, rendering traditional algorithms ineffective due to insufficient statistical significance.

To investigate the polarized side-scattered light patterns in low-scattering coefficient media, this paper presents a backward ray tracing method, achieving polarized Monte Carlo simulations for low-scattering media and verified by experiment.

The main contributions of this work are stated as follows:

(1) Retro-projection correction via backward ray tracing: analytically mapping photon exit coordinates onto the detector plane significantly reduces lateral positional errors, and the procedure has been experimentally validated.
(2) Research the variation law of side-scattering DoLP in ultra-low $\mu_{s}$ media: using a 532 nm wavelength sheet laser on 5 nm diameter TiO_2_ solutions (concentration 10^−3^–10^−2^ g/L, $\mu_{s}=$ 1.06 × 10^−6^–1.06 × 10^−5^ cm^−1^). We quantify the monotonic decrease in DoLP with increasing concentration, a trend that differs from the non-monotonic behavior reported in Ref. [20]. Our results therefore correct and refine the earlier findings in Ref. [20], demonstrating that DoLP declines steadily throughout the investigated concentration range.

The remainder of this paper is organized as follows. Section 2 describes the experimental apparatus and procedures: two side-scattering setups for 500 nm polystyrene microspheres ($\mu_{s}=$ 0.21 cm^−1^) and 5 nm TiO_2_ nanoparticle solutions ($\mu_{s}=$ 1.06 × 10^−6^–1.06 × 10^−5^ cm^−1^). This section also presents the polarized Monte Carlo framework and derives the backward ray tracing method. Section 3 compares simulated and measured side-scattering polarization profiles, quantitatively validating the algorithm and demonstrating the monotonic decrease in the DoLP with concentration. Section 4 concludes this paper and provides perspectives for future research.

## 2. Materials and Methods

### 2.1. Experimental Setup

**Figure 1:**
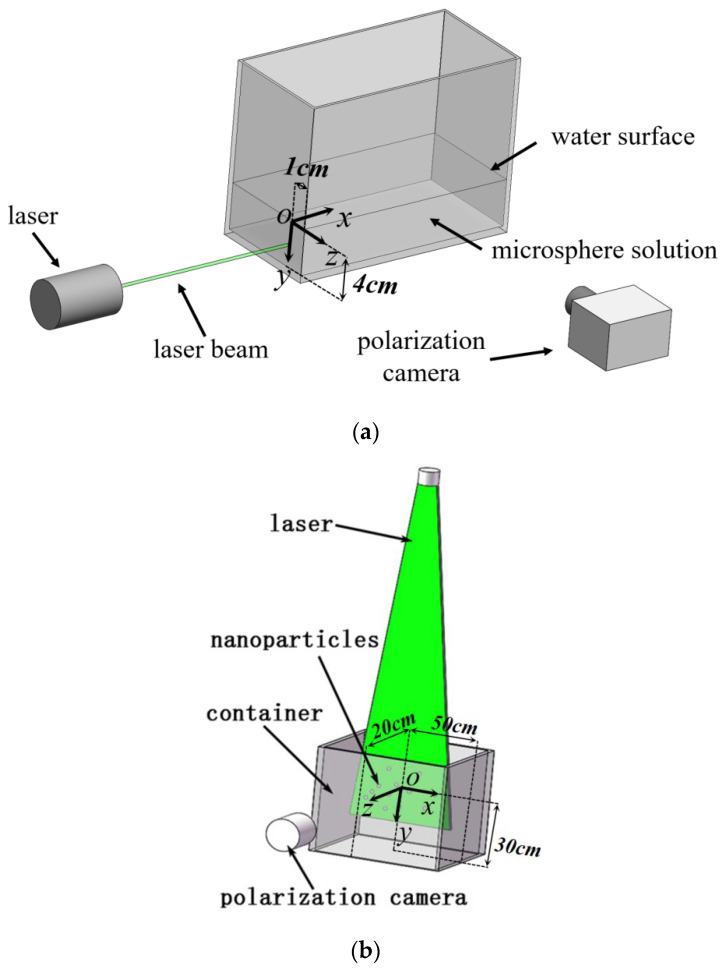
Schematic of the experimental setup employed in this study: (**a**) Experimental setup of Experiment 1. (**b**) Experimental setup of Experiment 2.

The configuration of experimental setups is depicted in Figure 1. The two experiments and Cartesian coordinate systems were established:

Experiment 1: A Neodymium-doped Yttrium Aluminum Garnet (Nd: YAG) laser operated at 532 nm wavelength with a beam diameter of 3 mm. The incident light was polarized parallel to the optical table. A dimension of 20 × 15 × 8 cm glass container with a 0.3 cm wall thickness was filled with a 500 nm diameter polystyrene microsphere solution (Langfei Biotechnology Co., Ltd., Shijiazhuang, China). The microsphere (density 1.05 g/cm^3^) had a refractive index of 1.59, and water exhibited a refractive index of 1.33. Microsphere concentrations were controlled at 7 × 10^−3^ g/L to achieve a low-scattering coefficient of $\mu_{s}=$ 0.21 cm^−1^.

Experiment 2: The same Nd: YAG laser was emitted and expanded into a sheet beam through a cylindrical mirror, and the thickness of the laser sheet was 1 mm. The incident light was polarized parallel to the polarization camera. A dimension of 100 × 40 × 50 cm glass container with a 0.4 cm wall thickness was filled with a 5 nm diameter TiO_2_ nanoparticle (density 4.23 g/cm^3^) solution, and the TiO_2_ nanoparticle had a refractive index of 2.60. Nanoparticle concentrations were controlled within the range of 10^−3^–10^−2^ g/L to achieve an ultra-low-scattering coefficient of $\mu_{s}=$ 1.06 × 10^−6^–1.06 × 10^−5^ cm^−1^.

A division-of-focal-plane (DoFP) polarization camera (FLIR company, Canada) was positioned next to the container for image capture. This configuration allows the camera to capture side-scattered light at four polarization angles (0°, 45°, 90°, and 135°) and subsequently compute the polarization parameters of the scattered light.

The DoFP polarization camera was calibrated prior to the experiments to ensure accurate Stokes parameter measurements. The calibration procedure included: (1) dark current correction, where the response offset *b* was measured under dark conditions and subtracted from all images; (2) responsivity calibration (flat-fielding), performed using an integrating sphere as a uniform light source—images were acquired at multiple intensity levels, and the detector responsivity *g* for each pixel was obtained via linear fitting, followed by a grouping correction method (grouped by the four micro-polarizer orientations) to reduce non-uniformity; (3) micro-polarizer orientation calibration, where an LED point source combined with a high-extinction-ratio linear polarizer mounted on a high-precision rotation stage was used to determine the actual orientation angles θ by fitting the cosine response curve; (4) crosstalk correction, applying the manufacturer’s native algorithm based on neighboring pixel interpolation. These calibrations minimized artifacts and ensured reliable polarization measurements.

### 2.2. Algorithm for Side-Scattering of Polarized Light in Media

**Figure 2:**
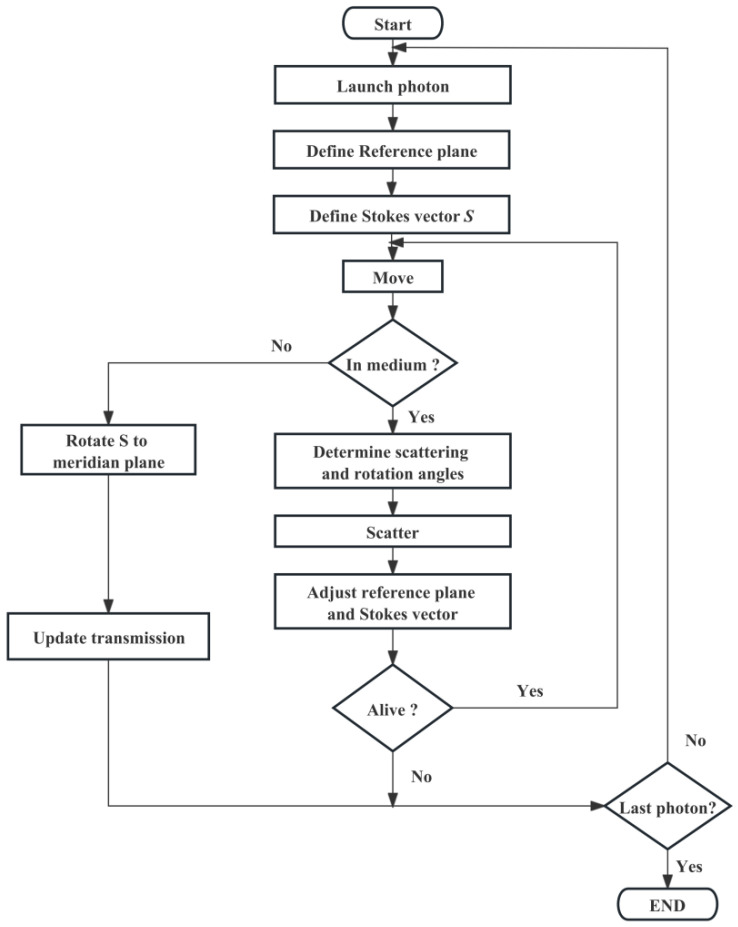
Flowchart depicting the simulation algorithm utilized for ensemble particle side scattering.

The algorithm’s flowchart is illustrated in Figure 2.

The specific procedure is outlined as follows: A photon is emitted in parallel to the *x*-axis within the scattering medium, determining its subsequent position ${\left[\begin{array}{ccc} x_{new} & y_{new} & z_{new} \end{array}\right]}^{T}$ based on its current location ${\left[\begin{array}{ccc} x & y & z \end{array}\right]}^{T}$, transmission direction ${\left[\begin{array}{ccc} u_{x} & u_{y} & u_{z} \end{array}\right]}^{T}$, and single scattering distance Δs.

The subsequent position ${\left[\begin{array}{ccc} x_{new} & y_{new} & z_{new} \end{array}\right]}^{T}$ is given by:

(1) $$\begin{array}{l} x_{new}=x+u_{x}\Delta s\\ y_{new}=y+u_{y}\Delta s\\ z_{new}=z+u_{z}\Delta s \end{array}$$

The expression for the single scattering distance Δs is given by:

(2) $$\Delta s=-\ln\left(\zeta \right)/\mu_{t}$$

where $\mu_{t}=\mu_{a}+\mu_{s}$; $\mu_{a}=q_{a}\rho A$ represents the absorption coefficient, $\mu_{s}=q_{s}\rho A$ represents the scattering coefficient, ζ is a pseudo-random number generated in the interval (0, 1], $q_{a}$ is the single-particle absorption coefficient, $q_{s}$ stands for the single-particle scattering coefficient, ρ signifies the particle concentration in the solution, and A denotes the cross-sectional area of the particle.

Following a single collision of a photon, assuming the incident photon Stokes vector is denoted as $S={\left[\begin{array}{cccc} I & Q & U & V \end{array}\right]}^{T}$, the Stokes vector after one scattering, $S_{new}$, can be represented as follows:

(3) $$S_{new}=R\left(-\gamma \right)M\left(\alpha \right)R\left(\beta \right)S$$

where the scattering angle α and the rotation angle β into the scattering plane are selected simultaneously by means of the rejection method applied to the phase function P(α,β). Three independent uniform deviates $\alpha_{rand}\in [0,\pi ]$, $\beta_{rand}\in [0,2\pi ]$ and $P_{rand}\in [0,1]$ are generated; the incident Stokes vector is denoted $S={\left[\begin{array}{cccc} I_{0} & Q_{0} & U_{0} & V_{0} \end{array}\right]}^{T}$. P(α,β) can be represented as follows:

(4) $$P(\alpha ,\beta )=S_{11}(\alpha )I_{0}+S_{12}(\alpha )[Q_{0}\cos(2\beta )+U_{0}\cos(2\beta )]$$

where $S_{11}(\alpha )$ and $S_{12}(\alpha )$ are the corresponding Mueller-matrix elements. $M\left(\alpha \right)$ is the Mueller matrix given by Mie scattering theory at the scattering angle α. $M\left(\alpha \right)$ can be expressed as [21]:

(5) $$M(\alpha )=\left(\begin{array}{cccc} S_{11}(\alpha ) & S_{12}(\alpha ) & 0 & 0\\ S_{12}(\alpha ) & S_{11}(\alpha ) & 0 & 0\\ 0 & 0 & S_{33}(\alpha ) & S_{34}(\alpha )\\ 0 & 0 & -S_{34}(\alpha ) & S_{33}(\alpha ) \end{array}\right)$$

$S_{11}(\alpha )$, $S_{12}(\alpha )$, $S_{33}(\alpha )$ and $S_{34}(\alpha )$ can be expressed as:

(6) $$\begin{array}{l} S_{11}=\frac{1}{2}\left(|s_{2}{|}^{2}+|s_{1}{|}^{2}\right),\;\;S_{12}=\frac{1}{2}\left(|s_{2}{|}^{2}-|s_{1}{|}^{2}\right),\\ S_{33}=\frac{1}{2}\left(s_{2}^{*}s_{1}+s_{2}s_{1}^{*}\right),\;\;S_{34}=\frac{i}{2}\left(s_{1}s_{2}^{*}-s_{2}s_{1}^{*}\right). \end{array}$$

The expression for $s_{1}$ and $s_{2}$ can be found in Ref. [21].

R(−γ) and R(β) are rotation transformation matrices generated at angles γ and β, respectively. R(β) can be represented as:

(7) $$R(\beta )=\left(\begin{array}{cccc} 0 & 0 & 0 & 0\\ \cos2\beta & \sin2\beta & 0 & 0\\ -\sin2\beta & \cos2\beta & 0 & 0\\ 0 & 0 & 0 & 1 \end{array}\right)$$

γ represents the angles at which the incident Stokes vector transitions from the reference plane to the scattering plane. The angle γ can be expressed as follows:

(8) $$\cos\gamma =\frac{-u_{z}+u_{z}^{\prime }\cos\alpha }{\pm \sqrt{\left(1-\cos^{2}\alpha )(1-{u_{z}^{\prime }}^{2}\right)}}$$

Transmission direction after a single scattering event $\left[\begin{array}{ccc} u_{x}^{\prime } & u_{y}^{\prime } & u_{z}^{\prime } \end{array}\right]$ can be represented as follows:

(9) $$\left\{\begin{array}{l} u_{x}^{\prime }=\sin\alpha (u_{x}u_{z}\cos\beta -u_{y}\sin\beta )/\sqrt{1-u_{z}^{2}}+u_{x}\cos\alpha \\ u_{y}^{\prime }=\sin\alpha (u_{y}u_{z}\cos\beta +u_{x}\sin\beta )/\sqrt{1-u_{z}^{2}}+u_{y}\cos\alpha \\ u_{z}^{\prime }=-\sin\alpha \cos\beta \sqrt{1-u_{z}^{2}}+u_{z}\cos\alpha \end{array}\right.$$

### 2.3. Backward Ray Tracing Algorithm

**Figure 3:**
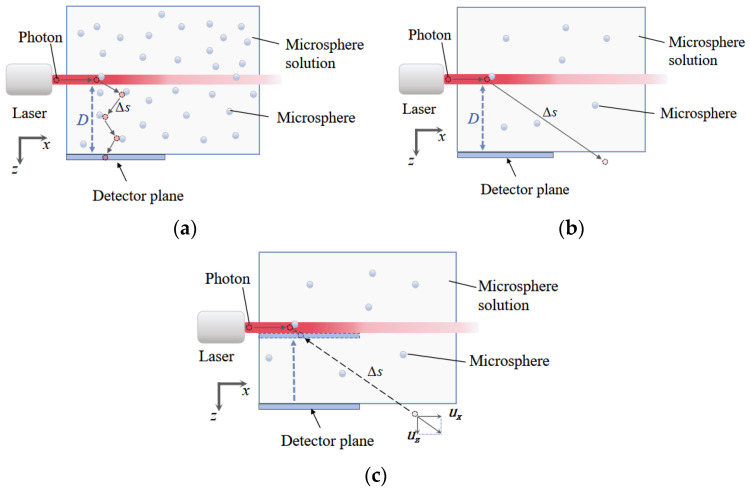
Schematic illustration of microsphere solution scenarios and backward ray tracing principle: (**a**) High-scattering coefficient regime. (**b**) Low-scattering coefficient regime. (**c**) The principle of the backward ray tracing.

To address positional deviations caused by photon exit positions that deviate from the detector plane in conventional polarized Monte Carlo methods, this study proposes a geometric projection correction algorithm based on backward ray tracing. To ensure a fair and consistent comparison with Ref. [16], we implemented its algorithm using the same finite detector geometry. The principle of the backward ray tracing method is shown in Figure 3. We use the equipment setups from Experiments 1 and 2 as an illustrative example.

We propose a criterion to define the “low-scattering coefficient” regime for side scattering. When the photon mean free path exceeds the lateral scattering distance *D* (i.e., the distance from the laser center to the medium boundary, which corresponds to the side distance from the laser center to the glass wall), it can be considered that single scattering predominantly occurs within the solution. For Experiment 1, *D* = 1 cm, and for Experiment 2, *D* = 20 cm.

(10) $$\Delta \bar{s}=\frac{1}{\mu_{s}}\geq D$$

Equivalently, rearranging the terms yields a criterion for the scattering coefficient:

(11) $$\mu_{s}\leq \frac{1}{D}$$

When the scattering coefficient satisfies Equation (11) (i.e., the scattering coefficient is less than the reciprocal of the lateral scattering distance *D*), the scattering coefficient is considered small, and the side scattering is in the “low-scattering coefficient” regime where conventional Monte Carlo methods fail.

Figure 3a depicts typical photon paths in a medium with a high-scattering coefficient. Photons emitted from the laser source undergo multiple scattering events within the solution. Due to the large scattering coefficient, the photon mean free path is relatively short ($\Delta \bar{s}$ ≤ 1 cm). In this example, *D* from the laser center to the detector plane is 1 cm. Consequently, the vast majority of photons travel only a short distance before detection, and their exit points are naturally concentrated near the detector plane, allowing for effective capture without additional correction.

Figure 3b illustrates the scenario in a medium with a low-scattering coefficient. The probability of photons interacting with scattering particles decreases sharply, resulting in a significant increase in the mean free path $\Delta \bar{s}$. For instance, in a medium with $\mu_{s}$ = 0.2 cm^−1^, the mean free path $\Delta \bar{s}$ can reach 5 cm, which is much larger than the detector distance *D* = 1 cm. This implies that, for over 80% of photons, their exit point after the first scattering event lies outside the detector plane, creating a “detection blind spot” in conventional Monte Carlo methods. To obtain statistically significant signals, the number of photons launched in the simulation would need to increase dramatically, up to the order of 10^11^, which is computationally infeasible and constitutes a major bottleneck for the practical application of traditional methods [16].

Figure 3c provides a detailed schematic of the photon path and the principle of geometric correction based on backward ray tracing. The black dashed line represents the projection of the photon path onto the detector plane. The core idea of this correction method is based on the following physical fact: in a low-scattering coefficient medium ($\mu_{s}\leq 1/D$), the photon mean free path is significantly larger than *D*. Therefore, the vast majority of photons undergo only a single scattering event (or zero scattering events). Taking Experiment 2 as an example, the lateral scattering distance *D* is determined to be 20 cm from Figure 1b. According to Equation (10), the photon mean free path $\Delta \bar{s}=1/\mu_{s}>3.5\times 10^{4}\;\mathrm{cm}\gg D=20\;\mathrm{cm}$, which is significantly larger than *D*. Simulation results further confirm the dominance of single scattering in the detected signal. Among the 10^9^ incident photons, at the lowest concentration (10^−3^ g/L), photons that have undergone two or more scattering events contribute only approximately 0.0422% of the total detected intensity, corresponding to a single scattering intensity proportion of 99.9578%. When the concentration is increased to 10^−2^ g/L, the contribution of multiply scattered photons rises to 0.3319%, still a small fraction, yet sufficient to cause a measurable decrease in the overall DoLP due to the vectorial cancelation effect described in Section 4.

For photons that experience single scattering, the scattering event can be considered to occur primarily on a plane parallel to the *y*-axis at *z* = *r*, where *r* is the radius of the laser beam.

When the *z*-coordinate of a photon’s exit position [*x_exit_*, *y_exit_*, *z_exit_*] in a simulation exceeds *D*, traditional methods would discard it as an invalid signal due to its deviation from the detector plane. To solve this geometric mismatch problem, the proposed algorithm retro-projects this photon’s exit point along its final propagation direction [*u_x_*, *u_y_*, *u_z_*] onto the detector plane, defined as the plane parallel to the *y*-axis at *z* = *r*. Through this operation, photons that would otherwise “escape” due to the excessively large mean free path are effectively remapped into the detection region, thereby correcting the geometric error.

(12) $$\begin{array}{l} x_{end}=x-\frac{u_{x}}{u_{z}}\left(z_{\mathrm{exit}}-r\right)\\ y_{end}=y-\frac{u_{y}}{u_{z}}\left(z_{\mathrm{exit}}-r\right) \end{array}$$

where *r* is the radius of the laser beam. In this study, the detector size used in the simulations strictly matches the field of view of the physical camera: in Experiment 1, the detector covers a 4 cm × 7 cm region in the xy-plane (at z = 1.5 mm); in Experiment 2, it covers a 4 cm × 7 cm region (at z = 0.5 mm).

In the extreme low-scattering coefficient limit ($\mu_{s}$ → 0), analytical single scattering models based on Mie theory and the Beer–Lambert attenuation law can, in principle, be employed for rapid calculations. However, such models are insufficient to fully describe the physical processes under our experimental conditions for several reasons. First, even at extremely low $\mu_{s}$ values, residual multiple scattering still contributes a non-negligible fraction of the signal and measurably affects the DoLP. Second, the side-detection geometry collects light from multiple paths and positions, which cannot be accurately represented by a single-particle scattering assumption. Furthermore, the experimentally observed decrease in DoLP with increasing concentration contradicts the near-unity DoLP expected from single-particle scattering, further underscoring the necessity of Monte Carlo simulations.

## 3. Result

### 3.1. Experiment 1: Low-Scattering Coefficient 500 nm Diameter Polystyrene Microsphere Solution

**Figure 4:**
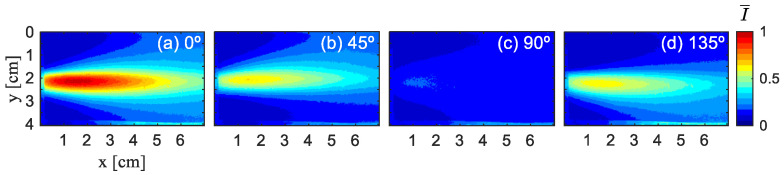
Experimental results for a 500 nm diameter polystyrene microsphere solution under 532 nm laser wavelength at $\mu_{s}=$ 0.21 cm^−1^. (**a**) 0° experiment, (**b**) 45° experiment, (**c**) 90° experiment, (**d**) 135° experiment.

**Figure 5:**
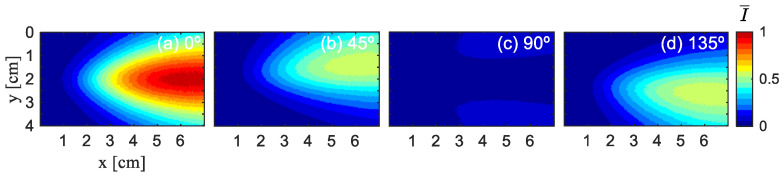
Simulation results for a 500 nm diameter polystyrene microsphere solution under 532 nm laser wavelength at $\mu_{s}=$ 0.21 cm^−1^ of Ref. [16]. (**a**) 0° simulation, (**b**) 45° simulation, (**c**) 90°simulation, (**d**) 135° simulation.

**Figure 6:**
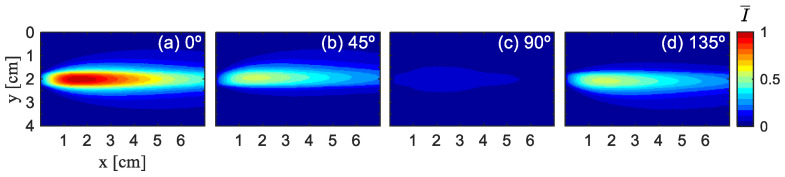
Simulation results for a 500 nm diameter polystyrene microsphere solution under 532 nm laser wavelength at $\mu_{s}=$ 0.21 cm^−1^ of this paper. (**a**) 0° simulation, (**b**) 45° simulation, (**c**) 90°simulation, (**d**) 135° simulation.

A total of 1 × 10^8^ photons were launched, with random numbers generated using the Mersenne Twister algorithm (seed 123456). Each photon was tracked until it escaped the medium (i.e., reached the boundaries at z = ±1 cm), at which point it was considered stationary and its Stokes vector recorded. Refraction at the glass boundaries was not considered in the simulation. Figure 4 illustrates the experimental results of Experiment 1, which display the distribution of normalized light intensity at different angles (0°, 45°, 90°, and 135°). Figure 5 and Figure 6 show the simulation results of Ref. [16] and this paper. To ensure consistency, all images of polarization directions undergo normalization based on the maximum light intensity value in four images, denoted as $\bar{I}$, normalized light intensity, and are visualized using the jet color map.

It can be observed that the overall trend and pattern distribution in Figure 6 are similar to those in Figure 4, indicating that our simulation results are basically consistent with the experimental results. Figure 5 shows the simulation results of Ref. [16]. It can be observed that, under the condition of a low-scattering coefficient, there is a significant difference between the simulation results presented in Ref. [16] and the experimental outcomes, and the former cannot effectively simulate the experimental situation.

**Figure 7:**
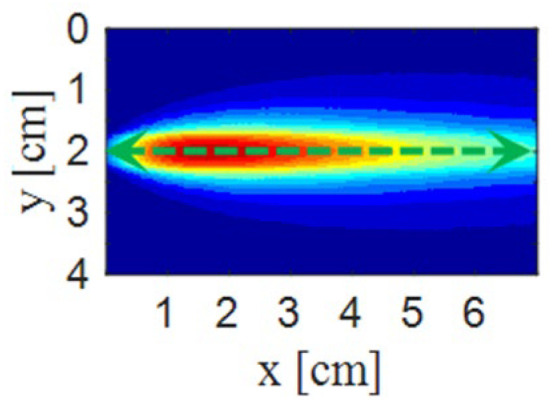
Schematic illustration of the sampling line.

To enable a more quantitative comparison of the simulated and experimental normalized light intensities, we perform sampling along the green line y = 2 cm, as shown in Figure 7. This line is selected because the results are symmetric at this y-position. Owing to the similar variation patterns of the normalized light intensity in all directions, Figure 4a and Figure 6a are chosen for analysis.

**Figure 8:**
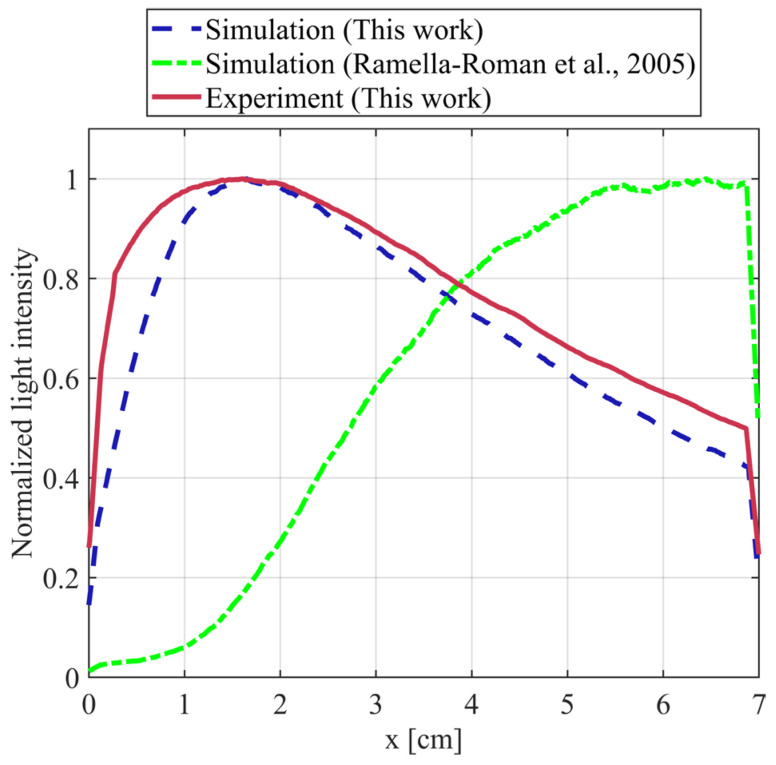
Normalized light intensity from sampling line data. Source: Ramella-Roman et al., 2005 [16].

Figure 8 shows the normalized light intensity profiles along the sampling line from x = 0 cm to x = 7 cm. Three curves are presented: the blue dashed line represents the simulation results obtained by our proposed method (this work), the green dotted line corresponds to the simulation results using the conventional Monte Carlo method described in Ref. [16], and the red solid line shows the experimental results (this work). The simulation results from our method exhibit a trend that closely matches the experimental data, with both curves peaking near x = 1.6 cm and showing good agreement in overall shape and magnitude. In contrast, the conventional method (Ref. [16]) fails to reproduce the experimental profile, significantly deviating from the measured intensity distribution.

To quantitatively evaluate the agreement between simulation and experiment, the coefficient of determination R^2^ is employed. R^2^ is calculated by selecting 30 evenly spaced data points from the sampling line.

(13) $$R^{2}=1-\frac{\sum_{i=1}^{n}{\left(y_{i}-{\hat{y}}_{i}\right)}^{2}}{\sum_{i=1}^{n}{\left(y_{i}-\bar{y}\right)}^{2}}$$

where *n* denotes the total number of observations. $y_{i}$ is the experimental value. ${\hat{y}}_{i}$ is the simulation value. $\bar{y}$ represents the sample mean of the experimental value.

**Figure 9:**
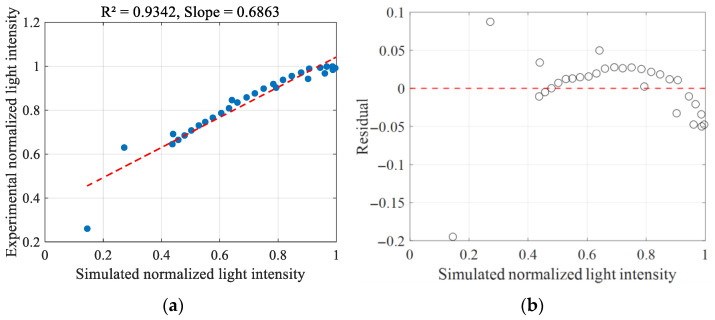
Correlation between the simulated and experimental normalized light intensity in Experiment 1 (this work): (**a**) Linear fit between simulated and experimental normalized light intensity (this work). (**b**) Residual plot of simulation and experimental normalized light intensity (this work).

Figure 9a illustrates the correlation between simulated and experimental normalized light intensity. The blue dots denote the data points, while the red dashed line indicates the linear fit. R^2^ value of 0.9342 signifies a strong linear correlation between the simulated and experimental results. The slope of 0.6863 implies that experimental values tend to be larger than simulated ones, likely due to background reflection of incident light during the experiment. Nevertheless, the high R^2^ value (0.9342) and the random distribution of residuals (Figure 9b) indicate that the simulation accurately captures the spatial distribution shape of the intensity. Furthermore, we calculated the R^2^ value between the simulation results of Ref. [16] and our experimental data, obtaining a value of 0.1510. This low R^2^ indicates that the conventional Monte Carlo method described in Ref. [16] fails to accurately reproduce the actual scattering behavior in low-scattering media.

Figure 9b illustrates the residual plot, which further elucidates the discrepancy between simulated and experimental values. Most residuals are tightly clustered around the zero line, with no apparent pattern or trend. This distribution of residuals supports the reliability of the simulation model. Despite the experimental values being slightly higher, the high R^2^ value and the random distribution of residuals collectively demonstrate that the simulation results are in good agreement with the experimental data. This suggests that the simulation model effectively captures the underlying physical phenomena.

The linear fit in Figure 8 shows a slope of 0.6863 between simulated and experimental values, indicating that experimental intensities are systematically higher than simulation results. This is likely attributable to background scattering present in the experiment, such as reflections from the glass container walls and residual ambient light, which add an approximately constant offset to the measured intensity. Ideally, this offset could be removed by measuring the background signal with pure water; however, due to shot-to-shot fluctuations in laser output intensity, obtaining a stable and reproducible background value for precise subtraction is challenging in practice. Therefore, background subtraction was not performed, and the raw comparison is presented.

### 3.2. Experiment 2: Ultra-Low-Scattering Coefficient 5 nm Diameter TiO_2_ Nanoparticle Solution

**Figure 10:**
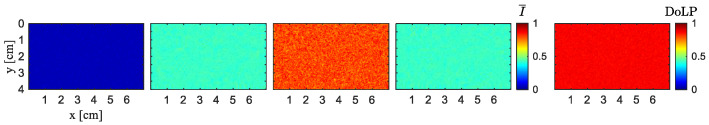
Experimental results for a 5 nm diameter TiO_2_ solution under 532 nm laser wavelength at $\mu_{s}=$ 1.06 × 10^−5^ cm^−1^.

**Figure 11:**
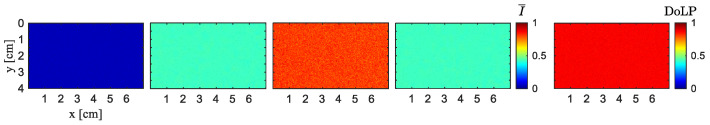
Simulation results for a 5 nm diameter TiO_2_ solution under 532 nm laser wavelength at $\mu_{s}=$ 1.06 × 10^−5^ cm^−1^ of this paper.

A total of 1 × 10^9^ photons were launched, with random numbers generated using the Mersenne Twister algorithm (seed 123456). Each photon was tracked until it escaped the medium (i.e., reached the boundaries at z = ±1 cm), at which point it was considered stationary and its Stokes vector recorded. Figure 10 and Figure 11, respectively, present the simulation and experimental results for Experiment 2 at a concentration of 0.01 g/L, $\mu_{s}=$ 1.06 × 10^−5^ cm^−1^. DoLP can be calculated using the following equation:

(14) $$\mathrm{DoLP}=\frac{\sqrt{S_{1}^{2}+S_{2}^{2}}}{S_{0}}$$

where S_0_, S_1_, and S_2_ denote the first three terms of the Stokes parameters. The Stokes parameters is formulated with light intensity, as represented by:

(15) $$\left[\begin{array}{c} S_{0}\\ S_{1}\\ S_{2} \end{array}\right]=\left[\begin{array}{l} I_{0}+I_{90}\\ I_{0}-I_{90}\\ I_{45}-I_{135} \end{array}\right]$$

In Figure 10 and Figure 11, the spatial distribution of normalized light intensity and DoLP from both experiment and simulation are presented. The simulation results show good consistency with the experimental results. The relatively uniform appearance of the light intensity images is likely due to the even distribution of nanoparticles within the medium. This uniform distribution ensures that light scatters consistently across the sample, leading to a homogeneous intensity pattern in the captured images. The DoLP images further complement these observations by showing corresponding polarization characteristics. The consistency between the simulation and experimental results validates the simulation model’s ability to accurately predict the light scattering and polarization properties under these experimental conditions, highlighting the model’s reliability and the influence of nanoparticle distribution on the optical properties observed.

**Figure 12:**
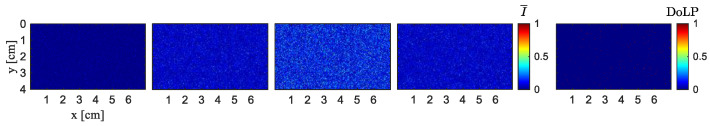
Simulation results for a 5 nm diameter TiO_2_ solution under 532 nm laser wavelength at $\mu_{s}=$ 1.06 × 10^−5^ cm^−1^ of Ref. [16].

Figure 12 shows the simulation results of Ref. [16]. It can be observed that, under the condition of an ultra-low-scattering coefficient, there is a significant difference between the simulation results presented in Ref. [16] and the experimental outcomes, and the former cannot effectively simulate the experimental situation.

**Figure 13:**
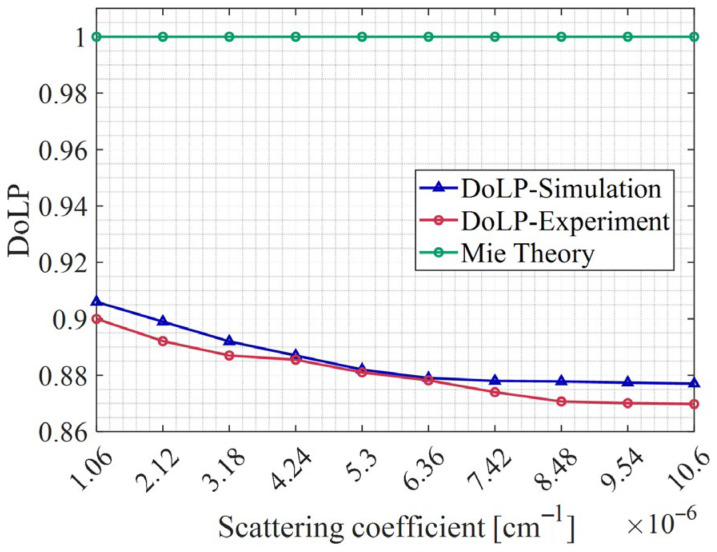
Relationship between DoLP and concentration.

Figure 13 shows the variation trend of DoLP with concentration, comparing the simulation and experimental results. Both the simulation and experimental results exhibit a similar monotonic decrease in DoLP as the concentration increases. For reference, the DoLP predicted by single-scattering Mie theory is also shown as a horizontal line at unity. This theoretical value is independent of concentration and remains constant (approximately 1). The stark contrast between the constant Mie prediction and the decreasing trend observed in both simulation and experiment clearly indicates that the depolarization cannot be captured by single scattering analytical models. The close agreement between our simulation and experimental data further demonstrates the accuracy and reliability of the proposed Monte Carlo method in capturing the concentration-dependent depolarization behavior. It is worth noting that conventional polarized Monte Carlo methods without the proposed geometric correction are unable to reproduce this trend, underscoring the superiority and necessity of our approach.

Unlike the findings of Ref. [20], which reported a non-monotonic DoLP variation over a wider concentration range, our results show a monotonic decrease in DoLP with concentration in the ultra-low-scattering regime. This discrepancy likely arises from the different concentration ranges and experimental geometries; Ref. [20] used a smaller container, which may enhance wall reflections and multiple scattering effects.

**Figure 14:**
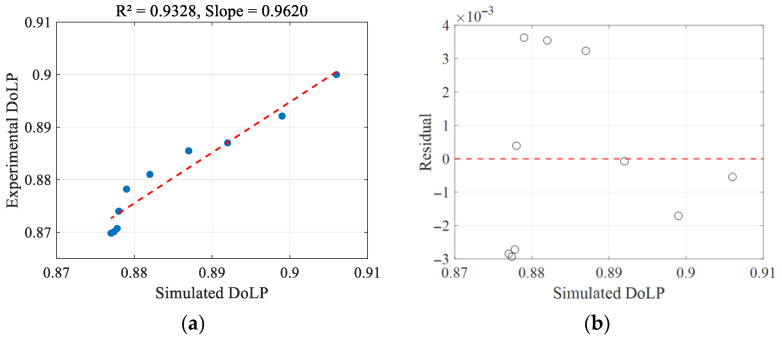
Correlation between the simulated and experimental normalized light intensity in Experiment 2: (**a**) linear fitting between simulation and experimental normalized light intensity; (**b**) residual plot of simulation and experimental normalized light intensity.

Figure 14a presents the linear fitting of DoLP between simulation and experiment. The high R^2^ value of 0.9328 and a slope of 0.9620 demonstrate a strong correlation between the simulated and experimental data, indicating the reliability of the simulation model in predicting DoLP variations with concentration. Figure 14b shows the residual plot of the simulated and experimental DoLP. The residuals are randomly distributed around the zero line with no obvious pattern, suggesting that the differences between the simulated and experimental values are within a reasonable range and further supporting the model’s accuracy.

## 4. Discussion

The backward-projection algorithm proposed in this study addresses the geometric mismatch problem inherent in conventional Monte Carlo simulations for low-scattering media. The experimental results presented in Section 3 demonstrate that our method accurately reproduces both the spatial distribution of scattered light and the polarization characteristics across two distinct particle systems covering low to ultra-low-scattering regimes. The high correlation coefficients (R^2^ > 0.93) between simulation and experiment confirm the validity of our approach under these conditions. In the ultra-low-scattering regime, the detected signal is dominated by single scattering. For single scattering, the DoLP is determined by the scattering angle and particle properties via the Mie scattering matrix. However, as concentration increases, the probability of photons undergoing two or more scattering events increases, albeit still small. Multiple scattering tends to depolarize the light because each scattering event randomizes the polarization state to some degree. The fraction of multiple scattered photons scales approximately with μs. Therefore, as $\mu_{s}$ increases (with increasing concentration), the multiple scattered fraction increases, leading to a monotonic decrease in the overall DoLP. Figure 13 shows that the multiply scattered fraction increases monotonically with concentration and that the DoLP of multiple scattered photons is significantly lower than that of singly scattered photons. Before discussing the broader implications, it is important to clarify the physical basis and underlying assumptions of this method.

According to the Stokes vector formalism, for incoherent superposition of light (e.g., photons arriving at the detector), the total polarization state is obtained by summing the Stokes vectors of individual photons [22].

(16) $$I_{\mathrm{total}}=\sum I_{i},\;\;\;Q_{\mathrm{total}}=\sum Q_{i},\;\;\;U_{\mathrm{total}}=\sum U_{i}$$

where ${\left[\begin{array}{cccc} I_{i} & Q_{i} & U_{i} & V_{i} \end{array}\right]}^{T}$ is the Stokes vector of the *i*-th photon. The DoLP of the combined light is then given by

(17) $$\mathrm{DoLP}=\frac{\sqrt{Q_{\mathrm{total}}^{2}+U_{\mathrm{total}}^{2}}}{I_{\mathrm{total}}}$$

This vectorial addition implies that, even if multiple scattered photons contribute only a small fraction to the total intensity, their polarization directions can significantly influence the overall DoLP. In particular, if the polarization directions of multiply scattered photons are orthogonal or opposite to the predominant direction of singly scattered photons, their Q and U components partially cancel those of the singly scattered photons, thereby reducing the magnitude of the resultant $\left[\begin{array}{cc} Q_{\mathrm{total}} & U_{\mathrm{total}} \end{array}\right]$ vector. This cancelation effect can produce a measurable decrease in the total DoLP despite the small intensity fraction, a phenomenon that highlights the higher sensitivity of polarimetric measurements compared to pure intensity measurements. This mechanism explains why trace multiple scattering must be accounted for in accurate simulations and why single scattering analytical models (e.g., Mie theory) are insufficient to describe the observed concentration-dependent depolarization.

Physical basis of the retro-projection: The retro-projection operation is not an arbitrary geometric manipulation but is derived from the principle of reciprocity in light transport. In low-scattering media where the scattering mean free path $1/\mu_{s}$ is significantly larger than the characteristic detector distance *D*, the probability of a photon undergoing multiple scattering events is extremely low. Under these conditions, the scattering event can be approximated as occurring on a plane near the laser beam center. The reciprocal nature of light propagation ensures that the mapping of photon exit points along their final propagation direction preserves the reversibility of photon paths.

Energy conservation and Stokes vector consistency: The retro-projection preserves energy conservation because it does not create or destroy photons—it simply maps the exit point of a photon that would otherwise be discarded onto the detector plane along its final propagation direction. The Stokes vector of the photon remains unchanged during this mapping, as no additional scattering or absorption events are introduced. This ensures that the polarization information carried by each photon is faithfully retained.

Connection to the radiative transfer equation: The radiative transfer equation describes the evolution of the specific intensity along ray paths. In our method, we track photons along their physical paths until they exit the medium. The retro-projection only affects the final position at which the photon is recorded, not its propagation history. This is equivalent to collecting photons at a virtual detector plane and then mathematically transforming their recorded positions based on the known propagation direction—a procedure consistent with the linearity of the radiative transfer equation.

Simplifying assumptions: It should be noted that the current backward-projection algorithm adopts a simplifying assumption, namely, that all photons projected onto the detector plane are considered detectable, without explicitly applying a solid-angle filter based on the finite aperture of the camera lens. This simplification is justified in the present study by the following facts: (1) single scattering overwhelmingly dominates, so the angular distribution of photons is relatively simple; (2) the agreement with experimental results (R^2^ > 0.93) further supports the method’s validity under the conditions studied. Nevertheless, we acknowledge that solid-angle and lens constraints are important in more general scenarios. Future work will incorporate a full acceptance function based on actual camera parameters to extend the method’s applicability.

The experimental validation presented in Section 3 demonstrates that, despite these simplifications, our method accurately reproduces both the spatial distribution of scattered light and the polarization characteristics across two distinct particle systems covering low to ultra-low-scattering regimes. The high correlation coefficients (R^2^ > 0.93) between simulation and experiment confirm that the geometric projection correction captures the essential physics under these conditions.

## 5. Conclusions

This paper addresses the challenge of simulating polarized side-scattered light in low-scattering coefficient media by proposing an enhanced polarized Monte Carlo algorithm that incorporates backward ray tracing and geometric projection correction. The method effectively resolves the geometric mismatch issue inherent in conventional simulations, where photon exit positions often deviate from the detector plane, thereby improving the accuracy of both intensity and polarization predictions. Experimental validation using 500 nm polystyrene microspheres and 5 nm TiO_2_ nanoparticles under 532 nm laser illumination demonstrates excellent agreement between simulated and measured results. The algorithm successfully captures the monotonic decrease in the degree of linear polarization with increasing particle concentration, a key trend for sensing applications. This work provides a simulation tool for designing optical sensors in ultra-low media and has direct applicability in nanoparticle concentration sensing and aerosol monitoring.

## Data Availability

Data underlying the results presented in this paper are not publicly available at this time but may be obtained from the authors upon reasonable request.
